# Potential Benefits and Limitations of *Hericium erinaceus* Supplementation

**DOI:** 10.3390/nu18213469

**Published:** 2026-10-22

**Authors:** Marina Barišić, Sunčana Včelik, Biljana Crevar, Tomislav Soldo, Maja Miškulin, Tihomir Kovač

**Affiliations:** 1 Faculty of Food Technology Osijek, Josip Juraj Strossmayer University of Osijek, Franje Kuhača 18, 31000 Osijek, Croatiamaja.miskulin@mefos.hr (M.M.); 2 Faculty of Tourism and Rural Development in Požega, Josip Juraj Strossmayer University of Osijek, Vukovarska 17, 34000 Požega, Croatia; 3 Faculty of Medicine Osijek, Josip Juraj Strossmayer University of Osijek, Josipa Huttlera 4, 31000 Osijek, Croatia

**Keywords:** *Hericium erinaceus*, dietary supplementation, cognitive function, neuroprotection, hericenones, erinacines, neuroplasticity

## Abstract

*Hericium erinaceus* (Lion’s Mane) is an edible and medicinal mushroom that has attracted increasing scientific interest as a dietary supplement because of its potential effects on cognitive function, neuroprotection, mental well-being and healthy ageing. Its biological activities have been primarily attributed to bioactive compounds such as polysaccharides, hericenones and erinacines, which may influence neurotrophic signalling, neuroplasticity, oxidative stress, inflammation and neuronal survival. This review examines the potential benefits and limitations of *H. erinaceus* supplementation, with emphasis on its bioactive compounds, mechanisms of action, cognitive and neuroprotective effects, mental-health-related outcomes, safety and product quality. Experimental studies provide evidence for neurotrophic, antioxidant, anti-inflammatory and neuroprotective activities, while clinical studies suggest possible benefits for cognitive outcomes, particularly information-processing speed, subjective stress and well-being. However, findings are inconsistent across study populations and outcomes, and evidence regarding neurodegenerative diseases is still largely derived from preclinical studies. Interpretation is further complicated by small sample sizes, short intervention periods, variability in supplement formulations, differences between fruiting-body-derived and mycelium-derived preparations and insufficient standardisation of bioactive compounds. Although *H. erinaceus* supplementation is generally well tolerated, its safety during prolonged use and optimal dosage remains unclear. Overall, *H. erinaceus* shows potential as a dietary supplement for supporting cognitive and neurological health, but further well-designed clinical trials involving larger populations, longer intervention periods and standardised, well-characterised preparations are needed to confirm its clinical effectiveness and establish reliable recommendations for safe supplementation.

## 1. Introduction

In recent years, interest has increased in dietary supplements and naturally derived compounds that may support cognitive function and neurological health. Among these, medicinal mushrooms have received significant scientific and commercial attention for their bioactive properties and potential health benefits. One of the most extensively studied medicinal mushrooms is *Hericium erinaceus*, known as Lion’s Mane mushroom. Traditionally used in Asian medicine, it has recently become popular as a dietary supplement [1]. In addition to being an edible mushroom, *H. erinaceus* is recognised for its rich content of biologically active compounds that contribute to its potential health benefits. Studies have shown that *H. erinaceus* contains both macromolecular and low-molecular-weight bioactive compounds. Major components include polysaccharides, proteins, terpenoids, cerebrosides, phenolic compounds and sterols. In addition, phytochemical research has identified a wider range of secondary metabolites within the *Hericium* genus, including alkaloids, resorcinols and other terpenoid compounds, which may also contribute to its reported biological activities [2]. These compounds are associated with various physiological activities, such as antioxidant activity, immune modulation, gastrointestinal protection and potential anticancer effects. In addition, *H. erinaceus* has drawn attention for its proposed neuroprotective properties. Certain bioactive compounds, particularly hericenones and erinacines, have been studied for their possible role in stimulating nerve growth factor (NGF) synthesis and supporting neuroplasticity, suggesting potential applications in the prevention and management of neurodegenerative diseases [3]. Despite promising results from preclinical and selected clinical studies, current evidence is limited and further research is needed to assess the efficacy, safety and long-term effects of *H. erinaceus* supplementation [1]. Several recent reviews have examined different aspects of *H. erinaceus*. Menon et al. [1] systematically reviewed the reported benefits, uses and potential side effects of *H. erinaceus* as a dietary supplement with an emphasis on the available clinical and preclinical evidence. Contato and Conte-Junior [3] provided a broader narrative review of the neuroprotective, antioxidant, anti-inflammatory and antimicrobial potential of the mushroom. More recently, Gurung et al. [4] reviewed the bioactive constituents of *H. erinaceus*, with particular emphasis on extraction strategies, health benefits and applications in functional foods. These reviews provide important overviews of the current evidence but differ in their primary focus and methodological approach. The present review focuses specifically on the potential benefits and limitations of *H. erinaceus* supplementation, integrating evidence on its major bioactive constituents and proposed mechanisms with findings from human clinical studies. Particular emphasis is placed on distinguishing fruiting-body-derived from mycelium-derived preparations, as differences in their bioactive compound profiles may influence their biological effects and complicate comparisons between studies. In addition, the review critically considers the extent to which mechanisms identified in isolated compounds, extracts, cellular models and animal studies can be translated into outcomes observed in humans. Factors that may influence the interpretation of supplementation studies, including product composition, standardisation, dosage, bioavailability, safety and the heterogeneity of the available clinical evidence, are also considered. By integrating these aspects, this review aims to provide a focused assessment of both the potential benefits and the current limitations of *H. erinaceus* supplementation.

Relevant scientific publications were identified primarily through searches of PubMed and Google Scholar, supplemented by screening the reference lists of relevant review and primary research articles. The literature search focused on publications from 2021 to 2026 addressing *Hericium erinaceus* supplementation, its bioactive compounds, biological activities, proposed mechanisms of action, safety and potential health effects. Search terms included combinations of “*Hericium erinaceus*”, “Lion’s Mane”, “supplementation”, “bioactive compounds”, “hericenones”, “erinacines”, “neuroprotection”, “cognition”, “neuroplasticity”, “antioxidant”, “anti-inflammatory” and “safety”.

Original research articles, clinical studies, preclinical studies and relevant review articles were considered based on their relevance to the scope of this narrative review. Publications outside the defined 2021–2026 period or those not directly relevant to the objectives of the review were excluded. Titles and abstracts were screened for relevance, followed by full-text assessment when appropriate. Reference lists of relevant publications were also examined to identify additional studies.

## 2. Bioactive Compounds and Mechanisms of Action

### 2.1. Bioactive Compounds

**Figure 1:**
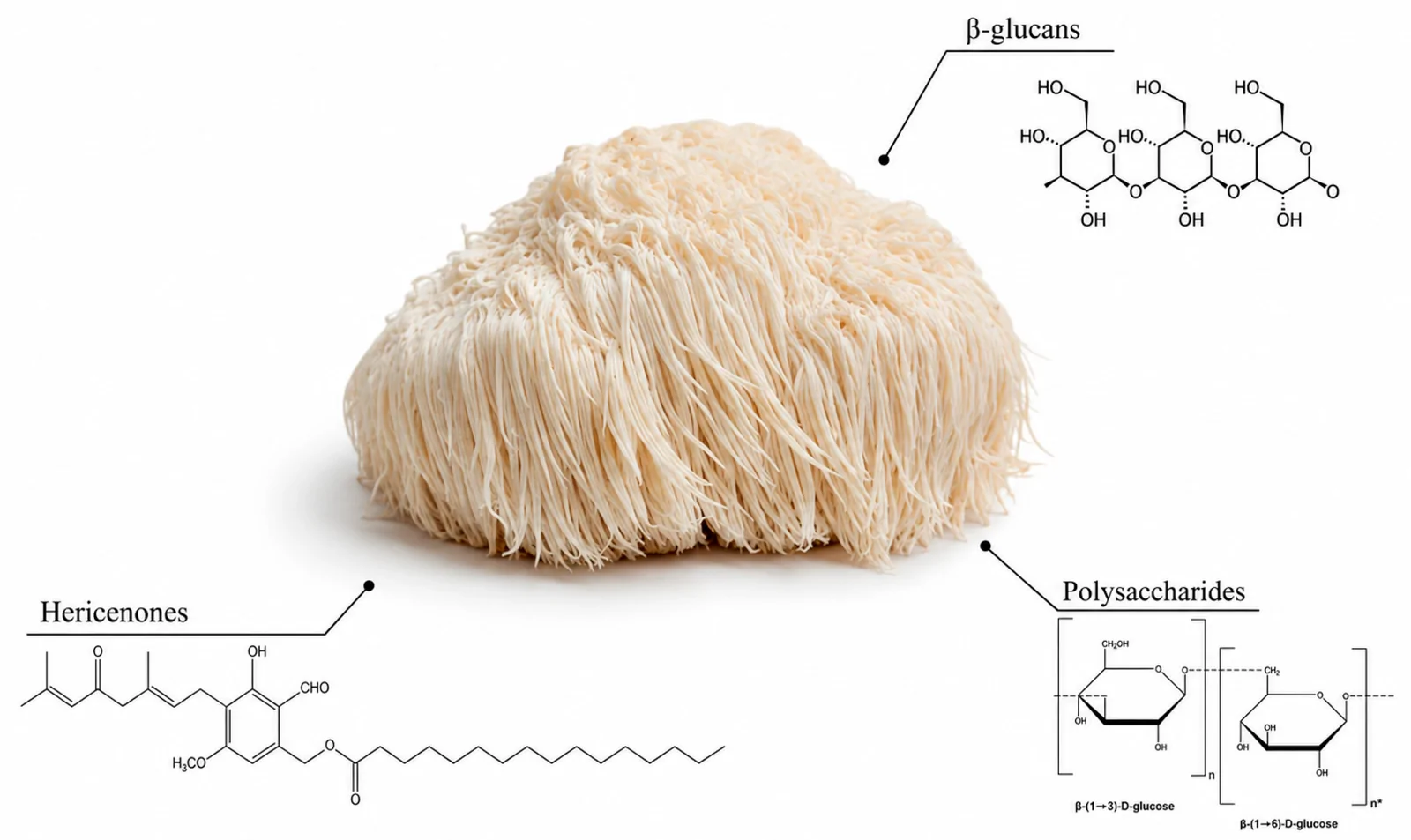
Selected bioactive compounds identified in the fruiting body of *Hericium erinaceus*.

The biological effects of *H. erinaceus* are attributed to various bioactive compounds that differ in chemical structure and physiological function. Among these, polysaccharides, hericenones and erinacines have received the most scientific attention for their potential contributions to antioxidant, anti-inflammatory and neuroprotective activities. These compounds may affect multiple biological pathways involved in neuronal growth, cellular protection and maintenance of cognitive function [3]. Selected bioactive compounds identified in the fruiting body of *H. erinaceus* are presented in Figure 1.

#### 2.1.1. Polysaccharides

Polysaccharides are among the main bioactive constituents of *H. erinaceus* and may contribute to its potential health-promoting properties. Among them, β-glucans are considered the most extensively studied polysaccharides and are regarded as key contributors to many of the mushroom’s biological activities. They have been widely studied for their antioxidant, immunomodulatory and anti-inflammatory activities. Their antioxidant effects may help reduce oxidative stress, while immunomodulatory activity may involve regulation of immune cell function and signalling pathways in host defence [5]. Recent studies also suggest that these compounds may support gastrointestinal health and influence gut microbiota composition, which could contribute to their broader biological effects [6]. However, differences in polysaccharide composition and extraction methods make comparisons between studies more difficult [3,7].

#### 2.1.2. Hericenones

Hericenones are a group of aromatic bioactive compounds that have attracted considerable scientific interest due to their potential neuroprotective properties. They are predominantly found in the fruiting body of *H. erinaceus* and are considered among the most relevant compounds associated with the mushroom’s neurological activity. Several structurally related hericenones have been identified in fruiting body material, including hericenones C–H [3,8]. Recent biosynthetic research has also provided further insight into how hericenone-related compounds are produced in the fungus. Iacovelli et al. [9] identified the first two enzymatic steps involved in the formation of these meroterpenoids. Interest in hericenones stems from their potential effects on the nervous system. Preclinical studies have associated hericenones C–H with stimulation of nerve growth factor (NGF) synthesis, with effects reported in both *in vitro* and *in vivo* experimental models [8]. A recent review further reported that hericenones C–E and H can stimulate NGF synthesis and that their activity differs between individual compounds [10]. NGF is a neurotrophic factor involved in neuronal growth, maintenance, differentiation and survival. Therefore, increased NGF synthesis may be relevant to neuronal regeneration and neuroplasticity, which is the ability of the nervous system to form new connections and adapt to physiological changes [8,10]. For this reason, hericenones have attracted interest in research on age-related cognitive decline and neurodegenerative diseases. Despite these promising findings, the biological relevance of hericenones in humans remains unclear. Most available evidence comes from *in vitro* experiments and animal studies, while clinical studies have mainly investigated whole-mushroom preparations rather than isolated hericenones. Therefore, it is difficult to determine the specific contribution of these compounds to the reported effects of *H. erinaceus* supplements. Further clinical studies using well-characterised preparations are needed to clarify their effects in humans [1].

#### 2.1.3. Erinacines

Erinacines are bioactive compounds classified as cyathane diterpenoids. They are primarily isolated from the mycelium of *H. erinaceus* and are considered one of the main groups of compounds responsible for its biological activity. Multiple erinacines have been identified, including erinacines A–S, and structural differences among them may influence their biological activity. Recent research has shown that erinacine production depends on tissue type and cultivation substrate, with mycelial tissue producing detectable erinacines, whereas fruiting body tissue generally contains little or none of these compounds [3,11]. Chemical studies have also isolated erinacines A, C and S from *H. erinaceus* mycelia, together with the newly identified compound erinacerin W, which belongs to a distinct erinacerin series [12]. Preclinical studies have investigated erinacines A, C and S in neuronal, glial and rodent models, with erinacine A being the most extensively studied [13]. *In vitro* studies have used erinacines at concentrations ranging from 0.01 ng/mL to 25 μg/mL, while animal studies have generally used oral doses of individual erinacines ranging from 2.6 to 30 mg/kg/day [13]. Scientific interest in erinacines has increased because of their potential effects on nervous system function. Preclinical research suggests that some erinacines, particularly erinacine A, may cross the blood–brain barrier and influence mechanisms associated with neuronal protection [13,14]. Erinacines have also been studied for their potential role in stimulating nerve growth factor (NGF), a neurotrophic factor involved in neuronal development, maintenance and survival. By influencing NGF-related pathways, these compounds may support neuronal regeneration and neuroplasticity, the nervous system’s ability to reorganise and form new neuronal connections [10,15]. In addition, experimental studies have associated erinacines with antioxidant and anti-inflammatory activities. Through effects on oxidative stress and inflammatory responses, these compounds may contribute to cellular protection and the maintenance of nervous system homeostasis [1,3]. Liu et al. [16] reported substantial differences in erinacine A content among *H. erinaceus* mycelial strains, indicating that strain selection may influence the composition of mycelial preparations.

### 2.2. Mechanisms of Action

The biological effects of *H. erinaceus* are attributed to several bioactive compounds, particularly hericenones, erinacines and polysaccharides. These compounds may influence multiple biological pathways that contribute to the mushroom’s potential health-promoting properties. The most extensively studied mechanisms include stimulation of nerve growth factor (NGF) synthesis, support for neuroplasticity and neuroprotection, antioxidant and anti-inflammatory activities [10,15].

#### 2.2.1. Stimulation of Nerve Growth Factor (NGF)

Nerve growth factor (NGF) is a neurotrophic protein that plays a crucial role in the growth, differentiation, maintenance and survival of neurons. It is especially important for the development and function of the central and peripheral nervous systems. Reduced NGF signalling has been associated with neuronal dysfunction and may contribute to neurodegenerative processes [10,15]. One of the most extensively studied mechanisms of *H. erinaceus* is its potential to influence NGF synthesis and neurotrophic signalling. Hericenones and erinacines have been investigated for their effects on NGF-related responses in preclinical models. Hericenones C, D, E and H have been reported to stimulate NGF synthesis, while erinacines A–F and H have also been studied for their NGF-stimulating effects [10].

In mouse astroglial cells, erinacines A, B and C increased NGF synthesis when tested at 1.0 mM, resulting in NGF levels of 250.1 ± 36.2, 129.7 ± 6.5 and 299.1 ± 59.6 pg/mL, respectively. Erinacines E and F, tested at 5.0 mM in the same experimental system, induced NGF levels of 105.0 ± 5.2 and 175.0 ± 5.2 pg/mL, respectively [10]. Erinacine D was also reported to stimulate NGF synthesis at 1.67 mM in rat astroglial cells, resulting in 141.5 ± 18.2 pg/mL NGF [10]. In 1321N1 human astrocytoma cells, erinacines A, B, C and E were reported to enhance neurotrophin production, as indicated by increased NGF mRNA expression, while erinacine C also increased BDNF mRNA expression [10]. Studies of hericenones C–E and H have further suggested that their effects on NGF synthesis and gene expression involve protein kinase A signalling [10]. These findings indicate that individual compounds may influence neurotrophic signalling through different experimental models and molecular responses. However, the available evidence is predominantly derived from preclinical models, and their contribution to the effects of whole *H. erinaceus* preparations used as supplements remains uncertain [10,13]. Most evidence for NGF stimulation has been obtained using isolated compounds or experimental *H. erinaceus* preparations in cellular and animal models [10,13]. Importantly, human studies have generally investigated whole-mushroom or enriched preparations rather than isolated hericenones or erinacines, making it difficult to determine the contribution of individual compounds to the effects observed following supplementation [1,13]. Differences in experimental models, concentrations and preparation composition also limit direct comparison between studies [10,13]. Thus, although NGF stimulation provides a plausible mechanism for the neurotrophic effects of *H. erinaceus*, current evidence is insufficient to establish a direct relationship between the NGF-stimulating activity of individual compounds and clinically relevant cognitive or neurological outcomes [1,10,13].

#### 2.2.2. Neuroplasticity and Neuroprotection

Neuroplasticity is the ability of the nervous system to modify and adapt its structure and function in response to internal and external stimuli. It includes the formation of new neuronal connections, remodelling of existing synapses and reorganisation of neural networks. These processes are important for learning, memory, cognitive performance and recovery after neuronal injury. Evidence from preclinical studies suggests that the neuroprotective effects of *H. erinaceus* may involve several interconnected neurotrophic signalling pathways. Erinacine A has been associated with activation of TrkA and downstream ERK1/2 signalling, while *H. erinaceus* mycelial extracts and erinacine A have also been linked to TrkA/TrkB-related signalling involving the PI3K/AKT pathway. CREB may act as a downstream transcription factor in these neurotrophic signalling pathways [13]. Erinacine C has been reported to increase BDNF expression in primary mixed glial and astrocytic cell models, while direct activation of CREB has been observed following erinacine C administration in a traumatic brain injury model [13]. In a mouse hippocampal neuronal cell model, *H. erinaceus* metabolites have also been associated with reduced caspase-3 activity, suggesting a potential anti-apoptotic effect [17]. Beyond these neurotrophic pathways, experimental studies indicate that *H. erinaceus* mycelium and its bioactive constituents may affect oligodendrocyte maturation, a process relevant to myelination and normal neuronal function [18].

**Figure 2:**
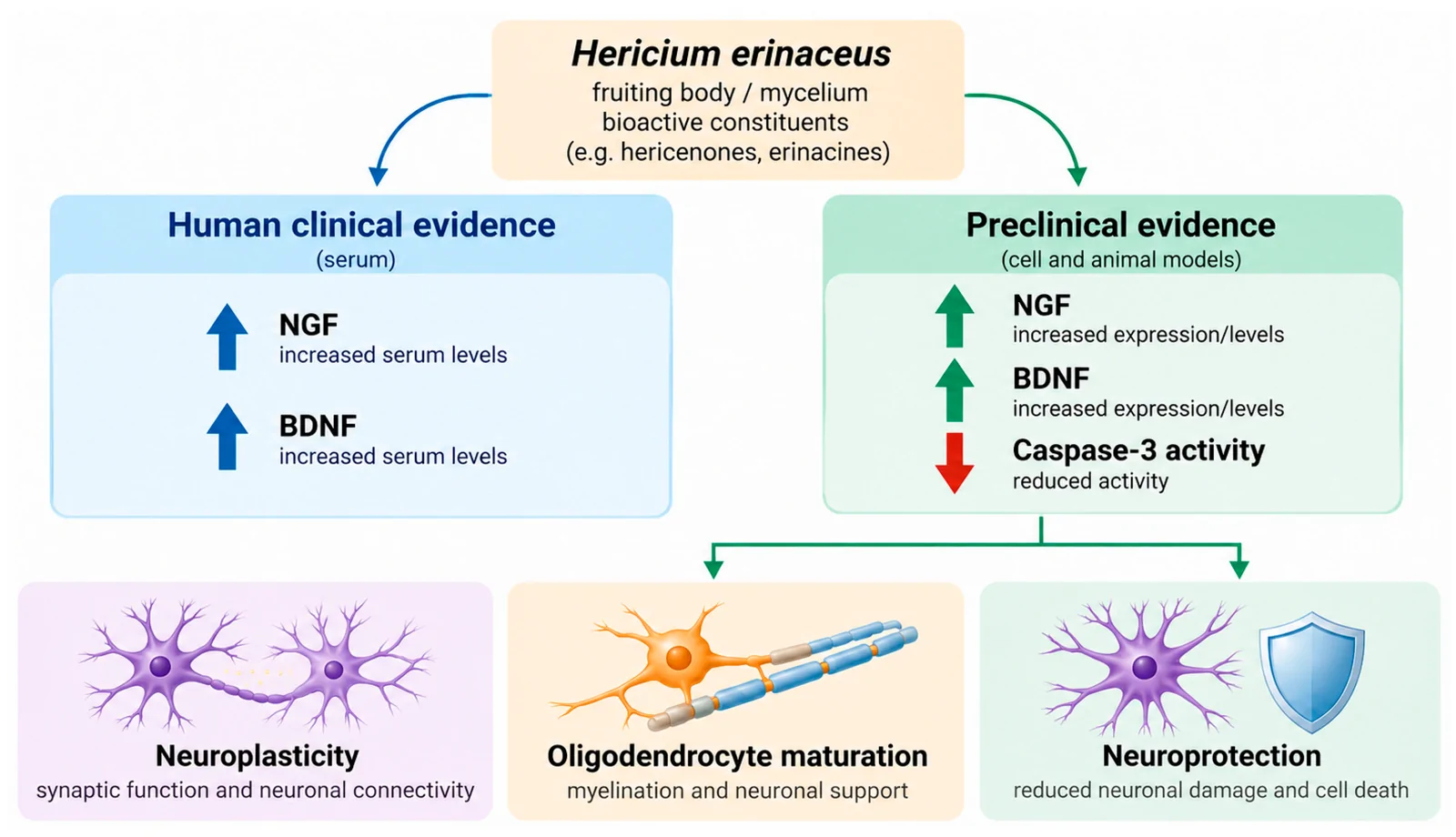
Neurotrophic and neuroprotective effects of *Hericium erinaceus* based on human and preclinical evidence. NGF, nerve growth factor; BDNF, brain-derived neurotrophic factor.

Nevertheless, these mechanisms have been investigated predominantly in preclinical models, and the contribution of individual erinacines to the effects observed with *H. erinaceus* preparations in humans remains uncertain [13,18]. Selected human and preclinical evidence on the neurotrophic and neuroprotective effects of *H. erinaceus* is summarised in Figure 2.

#### 2.2.3. Antioxidant and Anti-Inflammatory Effects

**Figure 3:**
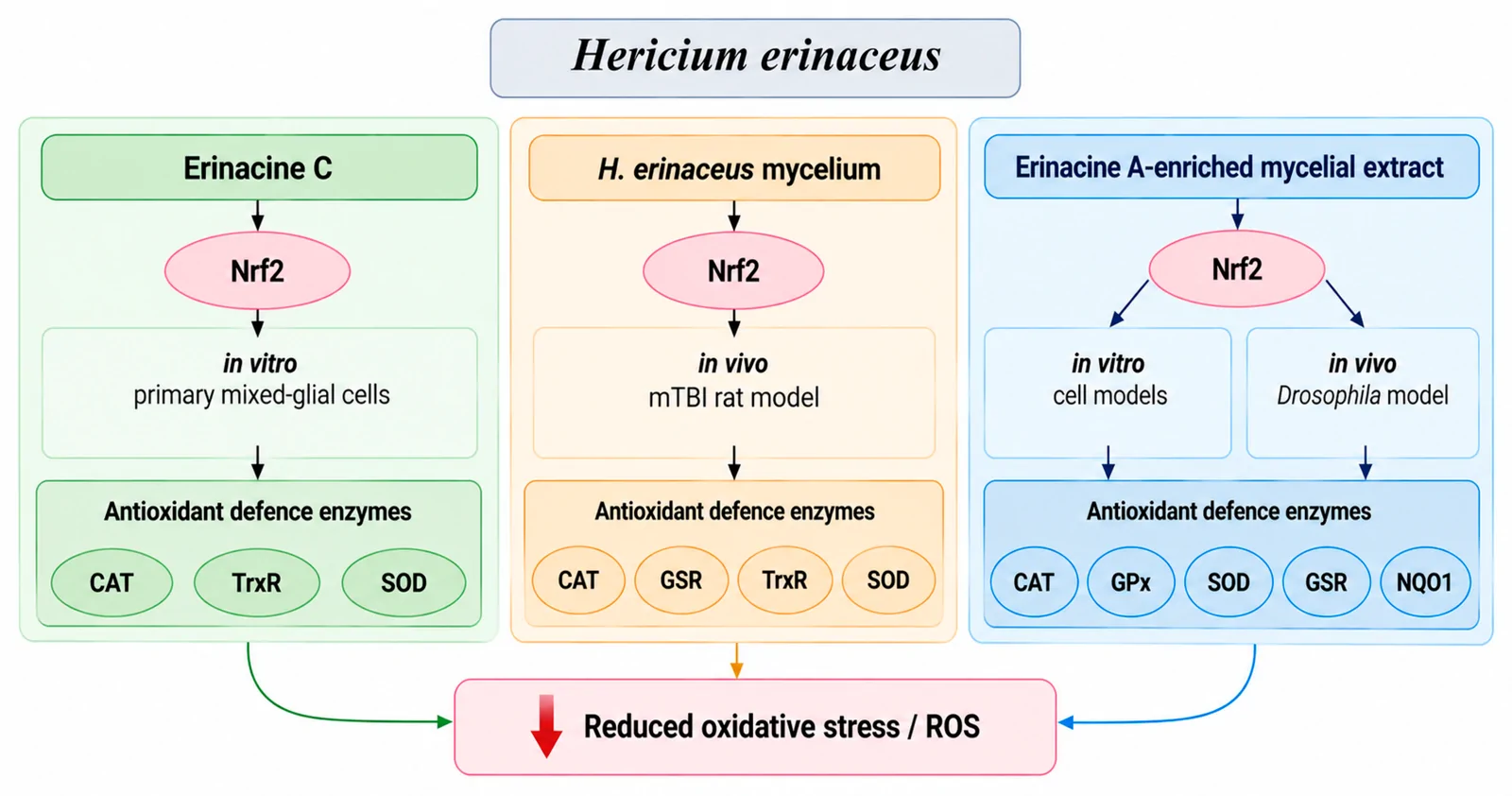
Nrf2-mediated antioxidant responses to *Hericium erinaceus* preparations and erinacines. Nrf2, nuclear factor erythroid 2-related factor 2; CAT, catalase; TrxR, thioredoxin reductase; SOD, superoxide dismutase; GSR, glutathione reductase; GPx, glutathione peroxidase; NQO1, NAD(P)H oxidoreductase 1; ROS, reactive oxygen species.

Oxidative stress refers to an imbalance between the production of reactive oxygen species (ROS) and the body’s antioxidant defence mechanisms. Excessive ROS accumulation can damage lipids, proteins and DNA, contributing to cellular dysfunction, ageing and the development of chronic diseases, including neurodegenerative disorders [1,14]. The antioxidant activity of *H. erinaceus* has been attributed to several bioactive compounds, including polysaccharides, hericenones and erinacines. These compounds may scavenge free radicals, support endogenous antioxidant defences and reduce oxidative damage at the cellular level. In experimental models, polysaccharides derived from the mushroom have been associated with reduced inflammatory responses and improved antioxidant status, including effects on pathways related to gastric mucosal protection [19]. In addition, preclinical evidence suggests that mycelium-derived preparations and erinacines may reduce oxidative stress and modulate neuroinflammatory pathways relevant to neuronal survival [13]. Preclinical studies further indicate that *H. erinaceus* preparations may modulate antioxidant defence through Nrf2-related mechanisms. In an *in vivo* model of mild traumatic brain injury, treatment with *H. erinaceus* mycelium and erinacine C was associated with increased expression of antioxidant defence enzymes, including catalase (CAT), glutathione reductase, thioredoxin reductase and superoxide dismutase (SOD). In primary mixed glial cells, erinacine C increased Nrf2 binding to the promoter regions of *CAT*, thioredoxin reductase and *SOD* genes, accompanied by increased expression of these genes [20]. In another experimental study, an erinacine A-enriched *H. erinaceus* mycelial ethanol extract increased Nrf2 activation and modulated the expression of antioxidant defence-related proteins, including CAT, glutathione peroxidase, SOD and NAD(P)H:quinone oxidoreductase 1 (NQO1), in cellular and *Drosophila* models. Nrf2 silencing attenuated the antioxidant responses induced by the extract, supporting the involvement of Nrf2 in these effects [21]. These findings indicate that modulation of Nrf2-associated antioxidant defence may contribute to the biological effects of *H. erinaceus*, although the available evidence remains predominantly preclinical. Selected preclinical findings on Nrf2-mediated antioxidant responses to *H. erinaceus* preparations and erinacines are summarised in Figure 3.

Anti-inflammatory activity is another proposed mechanism of *H. erinaceus*. Chronic inflammation and neuroinflammation are important contributors to neuronal dysfunction and the progression of neurodegenerative diseases. In a recent cell-based study, a hot-water extract reduced nitric oxide production and the expression and release of pro-inflammatory mediators in LPS-stimulated RAW 264.7 macrophages. The extract reduced the phosphorylation of IKK-α/β and NF-κB p65, as well as the phosphorylation of JAK1 and STAT3, while reducing IL-1β, IL-6 and TNF-α and increasing IL-10, indicating its potential to modulate inflammatory signalling pathways [22]. Diterpenoids isolated from *H. erinaceus* cultures have also shown anti-neuroinflammatory activity in experimental models [23]. NF-κB signalling has also been implicated in the effects of *H. erinaceus* polysaccharides. In RAW 264.7 macrophages, HEP-1, a polysaccharide fraction isolated from *H. erinaceus* fruiting bodies, promoted IκB-α degradation and nuclear translocation of NF-κB p65, which was associated with increased NF-κB transcriptional activity [24]. In contrast, HEP10, a low-molecular-weight polysaccharide isolated from *H. erinaceus* mycelia, reduced the phosphorylation of NF-κB p65 and IκB in LPS-stimulated RAW 264.7 macrophages. These effects were also observed in mice with DSS-induced colitis, where HEP10 reduced p65 and IκB phosphorylation in colon tissue and was associated with lower levels of several inflammatory markers, including TNF-α, IL-1β, IL-6, iNOS and COX-2 [25]. These findings suggest that *H. erinaceus* polysaccharides can affect NF-κB signalling, although the effects may differ depending on the preparation and experimental model.

**Figure 4:**
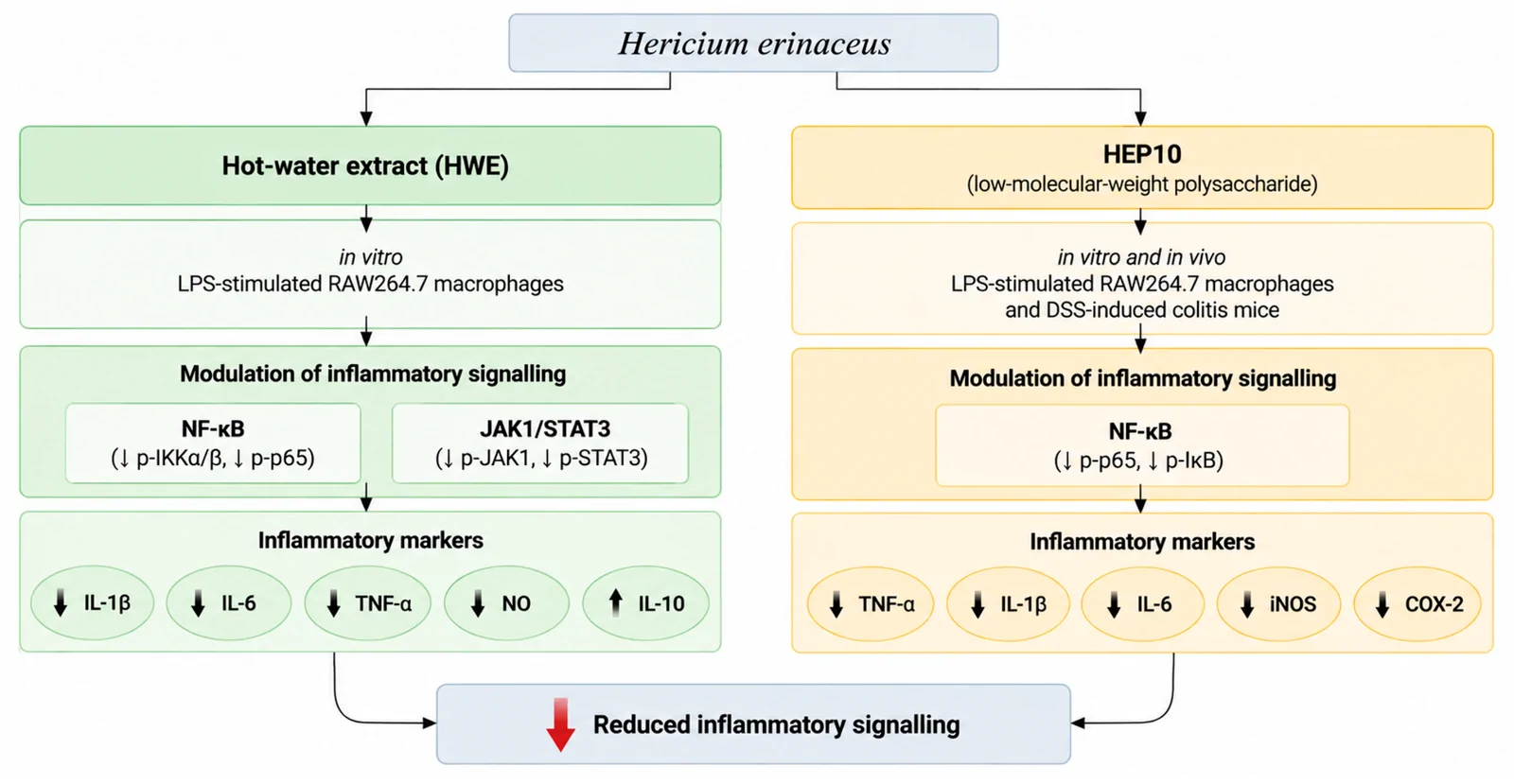
Anti-inflammatory effects and related molecular mechanisms of *Hericium erinaceus* preparations based on preclinical evidence. HWE, hot-water extract; HEP10, low-molecular-weight polysaccharide; NF-κB, nuclear factor kappa B; IκB, inhibitor of NF-κB; IKK, IκB kinase; p65, NF-κB p65 subunit; JAK1, Janus kinase 1; STAT3, signal transducer and activator of transcription 3; IL, interleukin; TNF-α, tumour necrosis factor alpha; NO, nitric oxide; iNOS, inducible nitric oxide synthase; COX-2, cyclooxygenase-2; DSS, dextran sulphate sodium.

Selected preclinical findings on the anti-inflammatory effects and related molecular mechanisms of *H. erinaceus* preparations are summarised in Figure 4. Together, these antioxidant and anti-inflammatory activities may contribute to the proposed neuroprotective effects of the mushroom and may be relevant to mechanisms implicated in age-related cognitive decline and neurodegenerative disorders [3,10].

Overall, the proposed mechanisms of action of *H. erinaceus* are supported mainly by preclinical studies using isolated compounds, extracts, cell models and experimental animals [1,12,13]. Although these studies provide evidence for neurotrophic, antioxidant and anti-inflammatory effects, their relevance to humans remains uncertain. Differences in fungal tissue, extraction methods, product composition and concentrations or doses used in experimental studies also make it difficult to compare findings and determine the contribution of individual compounds [3,13,14]. Therefore, these mechanistic findings should be considered supportive evidence rather than confirmation of clinical efficacy [1,12,13].

### 2.3. Bioavailability and Nanotechnology-Based Delivery Strategies

The bioavailability of bioactive compounds in *H. erinaceus* is an important consideration when evaluating their use as dietary supplements. Preclinical studies have shown that erinacine A can be absorbed following oral administration. In rats, its absolute oral bioavailability was estimated at 24.39%, and erinacine A was detected in the brain after oral administration, indicating that it can cross the blood–brain barrier [26]. Several recent studies have investigated nanotechnology-based delivery systems incorporating *H. erinaceus*-derived polysaccharides. Zhang et al. [27] developed sulfated *H. erinaceus* β-glucan–chitosan nanoparticles for resveratrol delivery. The nanoparticles showed good stability and sustained resveratrol release during simulated gastrointestinal digestion and enhanced anti-inflammatory effects in LPS-stimulated RAW264.7 macrophages. Although this approach did not involve hericenones or erinacines, it demonstrates the potential use of *H. erinaceus*-derived polysaccharides as components of nanodelivery systems. More recently, Zhao et al. [28] developed polyethyleneimine-modified PLGA nanoparticles containing *H. erinaceus* polysaccharides to address their limited oral bioavailability.

The optimised nanoparticles showed an encapsulation efficiency of 71.78% and stronger immunomodulatory effects in RAW 264.7 macrophages than free *H. erinaceus* polysaccharides and unmodified HEP-PLGA nanoparticles [28]. Qin et al. also developed macrophage membrane-coated PLGA nanoparticles containing *H. erinaceus* polysaccharides. These nanoparticles showed enhanced internalisation by macrophages and immunological effects in both *in vitro* and *in vivo* models [29]. To date, these delivery approaches have primarily been investigated for *H. erinaceus* polysaccharides, while studies on the nanoencapsulation or targeted delivery of individual compounds such as hericenones and erinacines remain limited. Moreover, the available evidence is predominantly derived from *in vitro* and animal models, and it remains unclear whether the improved delivery characteristics observed with these systems translate into increased systemic exposure or clinically relevant effects in humans. Further research is needed to determine whether nanotechnology-based delivery systems could improve the stability, absorption and bioavailability of these compounds and whether such approaches would be suitable for human use.

## 3. Potential Benefits of *Hericium erinaceus* Supplementation

The potential benefits of *H. erinaceus* supplementation have been investigated in relation to cognitive function, neuroprotection, mood, and other aspects of health. However, the available evidence varies according to the outcome studied, type of preparation, dose, population and study design [1,19]. The following sections summarise the main areas in which *H. erinaceus* supplementation has been evaluated. Human clinical evidence on *H. erinaceus* supplementation is summarised in Table 1, including study populations, product characteristics, dosage, intervention duration, main outcomes and key results. The studies differ substantially in participant characteristics, product composition, dosage, intervention duration and outcome measures, which should be considered when interpreting and comparing their findings.

The available human clinical studies are characterised by substantial heterogeneity in participant characteristics, product composition, dosage, intervention duration and outcome measures. Both fruiting body and mycelial preparations have been investigated, including erinacine A-enriched products, but differences in preparation and dosing make direct comparisons between studies difficult. Furthermore, the studies have assessed diverse outcomes, including specific cognitive functions, hearing, gut microbiota, metabolic flexibility, sleep and subjective well-being. This heterogeneity makes it difficult to determine whether the observed effects are attributable to *H. erinaceus* itself, specific bioactive compounds, or differences in preparation and study design. Previous reviews have likewise highlighted the variability and limitations of the available clinical evidence [1,37].

The relatively small sample sizes of several studies further limit the generalisability of the findings. Therefore, although some studies have reported beneficial effects on selected outcomes, the clinical evidence remains insufficient to establish consistent effects of *H. erinaceus* supplementation. Further well-designed, adequately powered, longer-term clinical trials using well-characterised and standardised preparations are warranted.

### 3.1. Cognitive Functions and Memory

Memory is one of the key cognitive functions involved in learning, storing and retrieving information.

With ageing, memory, attention, processing speed and executive function may decline, affecting daily functioning and quality of life. As a result, there is growing interest in dietary interventions that may support cognitive health during ageing [1,13]. Several clinical studies have examined the effects of *H. erinaceus* supplementation on cognitive performance. Current findings suggest possible benefits for selected cognitive outcomes, particularly information-processing speed, rather than consistent improvements across all cognitive domains. In a randomised, double-blind, placebo-controlled pilot study, Černelič Bizjak et al. [34] reported improved information-processing speed after eight weeks of supplementation with an erinacine A-enriched *H. erinaceus* product. The intervention was also associated with changes in gut microbiota composition and increased serum BDNF levels. However, these findings show an association and do not confirm the exact mechanism underlying the observed cognitive effects. Docherty et al. [33] reported an acute improvement in processing speed following a single dose, while chronic supplementation was associated with a trend towards reduced subjective stress in healthy young adults, although some cognitive outcomes did not change. In contrast, Surendran et al. [35] found no significant overall effect on cognition or mood after a single dose of a standardised fruiting-body extract in healthy younger adults. However, participants performed better on one psychomotor task, suggesting that acute effects may be limited to specific tasks. Preclinical evidence supports the potential effects of the mushroom on memory. In a zebrafish model of scopolamine-induced cognitive impairment, an ethanolic extract improved memory-related behavioural outcomes and was associated with reduced oxidative stress and changes in cholinergic markers [38]. Overall, current clinical studies suggest that *H. erinaceus* may influence selected aspects of cognitive performance. However, the results are not consistent across all cognitive domains or study populations. Differences in participant characteristics, product composition, dose, intervention duration and outcome measures may partly explain these inconsistencies [1,37].

### 3.2. Neuroprotection and Healthy Ageing

Ageing is associated with gradual changes in the nervous system, including increased oxidative stress, inflammation, and a higher risk of neurodegenerative disorders. These changes may contribute to declines in cognitive and neurological function. Therefore, there is growing interest in natural compounds that may help protect neurons and support healthy brain ageing [39]. Experimental studies suggest that *H. erinaceus* may have neuroprotective effects through several mechanisms. Its bioactive compounds and extracts have been associated with improved neuronal survival, reduced oxidative and inflammatory damage, and support of neurotrophic signalling. These effects may help maintain normal neuronal function during ageing [10]. In a mouse model of ageing, Roda et al. [39] found that long-term supplementation with an ergothioneine-rich *H. erinaceus* primordium extract prevented a decline in recognition memory. The supplemented mice also showed lower markers of oxidative stress and inflammation in the hippocampus. In another experimental study, a standardised *H. erinaceus* extract improved neuronal viability and reduced apoptosis in models of scopolamine-induced neurotoxicity.

It was also associated with increased acetylcholine levels and reduced acetylcholinesterase activity, suggesting possible effects on cholinergic signalling [40]. However, these findings are based on different experimental models and *H. erinaceus* preparations and the doses and exposure conditions used in these studies may not directly correspond to those achieved through dietary supplementation in humans. The contribution of individual compounds to the observed effects also remains difficult to determine. Overall, these findings suggest that *H. erinaceus* may affect several processes related to healthy brain ageing, including oxidative stress, inflammation, neuronal survival and cholinergic function. Nevertheless, most available evidence comes from cell and animal studies, and the effects in humans remain unclear [10,39,40].

#### 3.2.1. Alzheimer’s Disease

Alzheimer’s disease (AD) is the most common form of dementia, characterised by progressive cognitive decline, memory impairment and the accumulation of amyloid-β plaques and neurofibrillary tangles in the brain. These pathological changes are associated with neuronal dysfunction, synaptic loss and progressive neurodegeneration, ultimately leading to severe impairment of cognitive and daily functioning. In addition to pharmacological treatment, there is growing interest in lifestyle and nutritional approaches that may support cognitive health in people with mild cognitive impairment or early AD. For example, a multidomain lifestyle intervention including dietary changes, exercise, stress management, social support and supplements that included *H. erinaceus* was associated with favourable changes in cognitive and disease-related outcomes. However, because the intervention included several components, the specific contribution of the mushroom cannot be determined [41]. Because of its proposed neuroprotective properties, *H. erinaceus* has attracted considerable attention as a potential nutritional strategy for supporting cognitive health in the context of Alzheimer’s disease. Experimental evidence suggests that bioactive compounds in the mushroom may reduce amyloid-β accumulation, attenuate neuroinflammation and protect neurons from oxidative damage. Additionally, these compounds may support neuronal survival and help maintain cognitive function by influencing pathways involved in neurodegeneration [13,14]. Particular attention has been given to erinacine A, a cyathane diterpenoid isolated from the mycelium of *H. erinaceus*. This compound has attracted considerable interest because it can cross the blood–brain barrier and exert multiple neuroprotective effects. Studies suggest that erinacine A may stimulate the synthesis of neurotrophic factors, particularly nerve growth factor (NGF), which plays an essential role in neuronal survival, regeneration and maintenance. Experimental studies have associated erinacine A with effects on amyloid-β accumulation, neuroinflammatory responses and antioxidant pathways. In animal and cellular models, erinacine A has also been associated with modulation of microglial and astrocytic activation. However, these findings are derived predominantly from preclinical research and their relevance to human Alzheimer’s disease remains uncertain [13,14]. The available evidence comes from isolated compounds, enriched preparations and different animal and cellular models, making the findings difficult to compare directly. Although erinacine A has been shown to reach the brain and affect neurotrophic and inflammatory pathways in experimental models, it is not clear whether similar effects occur at comparable concentrations after oral supplementation in humans. Therefore, these findings support further research, but they do not yet show that *H. erinaceus* is effective in the treatment of Alzheimer’s disease. Animal studies have also reported improvements in learning and memory, together with reduced oxidative stress and inflammation following supplementation with *H. erinaceus* extracts. Furthermore, modulation of the gut microenvironment has been proposed as an additional mechanism contributing to the observed neuroprotective effects. For example, ergothioneine-rich extracts of *H. erinaceus* improved cognitive performance and were associated with reduced Alzheimer’s disease-related pathological changes in experimental models, together with effects on oxidative stress, inflammation and gut microbiota. Although current findings are encouraging, most available evidence comes from preclinical studies. Therefore, further well-designed clinical trials are needed to determine whether the beneficial effects observed in experimental models can be translated into effective therapeutic strategies for patients with Alzheimer’s disease [42].

#### 3.2.2. Parkinson’s Disease

Parkinson’s disease (PD) is a progressive disorder of the nervous system. It develops when dopamine-producing neurons in a brain region called the substantia nigra gradually die. This results in lower dopamine levels and causes symptoms such as tremor, muscle stiffness, slowed movement and balance problems. Many people with PD also experience cognitive difficulties, sleep disturbances, anxiety or depression. Experimental studies suggest that *H. erinaceus* may be relevant to PD because of its proposed neuroprotective and anti-inflammatory effects. Its bioactive compounds may help protect neurons, reduce oxidative stress and influence inflammatory processes involved in disease progression. In experimental models, these compounds have been associated with better survival of dopamine-producing neurons and reduced cellular damage [43]. Inflammation in the brain is also considered important in PD. Long-term activation of brain immune cells, such as microglia and astrocytes, can contribute to neuronal damage. In cell and animal models, erinacine A reduced the activation of these cells and protected dopamine-producing neurons from inflammation-related cell death [44]. Mushroom compounds such as ergothioneine may also help reduce oxidative damage and protect cells from stress. These properties may be relevant to neurodegenerative diseases, although current evidence is still mainly based on experimental studies [45]. Overall, the available findings are promising, but they do not yet confirm clinical benefits of *H. erinaceus* in people with Parkinson’s disease.

### 3.3. Potential Effects on Mental Health

In addition to its potential effects on cognitive function and neuroprotection, *H. erinaceus* has attracted attention for its possible role in mental health. Anxiety, depression and chronic stress can negatively affect emotional well-being, cognitive performance, and quality of life. Anxiety is characterised by excessive worry and nervousness, whereas depression is commonly associated with persistent sadness, loss of interest, reduced motivation, and difficulties in daily functioning.

Chronic stress may further worsen these conditions by affecting neuroendocrine regulation and increasing vulnerability to psychological distress. Several mechanisms may explain the potential effects of *H. erinaceus* on mental health. Neurotrophic factors such as nerve growth factor (NGF) and brain-derived neurotrophic factor (BDNF) are involved in neuronal survival, synaptic plasticity and emotional regulation. Reduced neurotrophic support, chronic inflammation and oxidative stress have all been associated with mood-related disorders. Through its neurotrophic, antioxidant and anti-inflammatory properties, *H. erinaceus* may support normal brain function. Another possible mechanism involves the gut–brain axis, a two-way communication system between the gastrointestinal tract and the central nervous system. Since changes in gut microbiota have been linked to anxiety, depression, and stress-related disorders, modulation of the gut microbiome may also be relevant [46]. Clinical evidence on mental-health-related outcomes is still limited. Some studies have reported improvements in subjective stress or selected measures of well-being after *H. erinaceus* supplementation [33]. However, findings are not consistent across all studies. For example, Surendran et al. [35] found no significant overall effect on mood after a single dose of a standardised *H. erinaceus* extract in healthy younger adults. These differences may be related to supplementation duration, dose, preparation type and participant characteristics. The available studies have mainly examined healthy participants and subjective measures of stress or mood rather than clinically diagnosed anxiety or depression, which limits conclusions about the effects of *H. erinaceus* in people with these conditions. Overall, current findings suggest that *H. erinaceus* may have potential to support selected mental-health-related outcomes, particularly stress-related measures. However, the available clinical evidence is limited and does not yet establish consistent effects on anxiety or depression [1,46].

## 4. Limitations of *Hericium erinaceus* Supplementation

The reported benefits of *H. erinaceus* should be interpreted with caution because the available evidence has several important limitations. Although experimental and clinical studies have reported promising findings, results are not consistent across studies or across cognitive outcomes. For example, Grozier et al. [31] found no significant improvement in cognitive performance after four weeks of supplementation in healthy college-aged adults. This variability may be related to differences in participant characteristics, supplement type and dose, intervention duration and outcome assessment methods. Additionally, variation in the composition and standardisation of commercial products makes it difficult to compare findings across studies. The following sections discuss the main limitations relevant to evaluating the potential benefits and long-term use of *H. erinaceus* supplements [1,35].

### 4.1. Lack of Clinical Evidence

Current reviews indicate that most evidence on *H. erinaceus* is still based on *in vitro* experiments and animal studies, while well-designed human clinical trials remain limited. Therefore, the promising biological effects observed in experimental models should be interpreted with caution when considering their potential relevance to humans [1,38]. A systematic review by Opanuga and Hossain [47] concluded that more robust and standardised human clinical trials are needed to confirm the potential health benefits of this mushroom. A similar limitation has been noted in sports-related research. Although potential effects on oxidative stress, glucose metabolism and cognitive function may be relevant to athletic performance, direct evidence for improvements in endurance, strength or recovery remains insufficient [48]. This limitation is also reflected in a review by Skubel et al. [49], which discussed the potential use of *H. erinaceus* in cognitive and depressive disorders, Alzheimer’s disease and post-traumatic brain injury, but noted that most of the cited evidence was derived from animal models. This gap is especially evident in research on neurodegenerative diseases. In a narrative review focused on Alzheimer’s disease, Cornford and Charnley [50] identified only three randomised clinical trials in humans, compared with thirteen animal-model studies. The authors emphasised that the small number of human studies, limited sample sizes and differences in intervention protocols restrict the reliability and generalisability of the available findings. Variations in the chemical composition and standardisation of the preparations used in these studies further complicate comparisons and make it difficult to determine whether the reported effects can be attributed to specific bioactive compounds or to the mushroom preparation as a whole. Similarly, Brandalise et al. [43] highlighted the need for more rigorous clinical research before *H. erinaceus* can be considered a clinically supported intervention for neurodegenerative conditions. Evidence is also lacking for more severe psychiatric conditions. Although its possible effects on inflammation, oxidative stress and neurogenesis have led to interest in its use as an adjunctive approach in chronic or treatment-resistant schizophrenia, this hypothesis has not yet been tested in clinical trials [51]. Overall, differences in participant characteristics, sample size, supplementation duration, product composition and outcome measures make it difficult to draw firm conclusions about clinical effectiveness. Larger randomised, placebo-controlled trials using standardised products and longer follow-up periods are needed to determine whether the promising effects observed in experimental studies can translate into clinically meaningful benefits for humans [1,47].

### 4.2. Lack of Standardisation of Supplements

A major challenge in evaluating *H. erinaceus* supplements is the lack of uniformity among commercial products. These may contain fruiting-body powder, mycelial biomass, extracts or combinations of these materials. Hericenones are mainly associated with the fruiting body, while erinacines are predominantly produced by the mycelium. Therefore, preparations from different sources may have substantially different chemical profiles and should not be assumed to produce equivalent biological effects [3]. Product composition may also vary according to fungal strain, cultivation substrate, growth conditions, harvesting stage, extraction procedure and storage conditions. For example, Tachabenjarong et al. [52] reported that fruiting bodies harvested at different growth periods showed differences in ergosterol, hericenone C, hericene A and antioxidant activity. This indicates that the harvesting stage may influence both the chemical profile and biological properties of the final product. As a result, the concentrations of hericenones, erinacines, polysaccharides, ergothioneine and other relevant constituents can differ considerably between products. Liu et al. [16] also reported substantial differences in erinacine A content among mycelial strains. Recent *in vitro* findings further highlight this issue: mycelial and fruiting-body preparations produced different cytokine responses in human immune cells, indicating that tissue source and extraction method may substantially influence biological activity [10]. This variability makes it difficult to compare clinical studies, identify the preparation responsible for a particular effect or establish evidence-based dosage recommendations. Analytical methods are therefore essential for confirming product identity and measuring concentrations of relevant compounds. Tang et al. [53] developed and validated a UHPLC-UV method for the simultaneous quantification of hericenones, hericenes, erinacines and ergosterol in raw materials and finished products. Similarly, Naumoska et al. [54] emphasised the importance of reliable procedures for the isolation, purification and quantification of individual compounds such as erinacine A. However, measuring the concentration of a bioactive compound in the final product does not necessarily reflect its availability in the body after supplementation. Kuo et al. [55] identified five common liver metabolites of erinacine A and reported its rapid metabolism in rat and human liver models. This suggests that standardisation should consider not only the initial concentration of a compound but also its bioavailability and metabolic fate after ingestion. The value of detailed product characterisation is also illustrated by Priori et al. [56], who investigated a standardised ethanol–water extract, referred to as He1, in elderly mice. Although this was a preclinical study, clearly defining the preparation allowed a more reliable interpretation of the observed effects. In addition, differences in product labelling and regulatory classification may affect consumer understanding and further limit product comparability across markets. Future clinical trials should therefore report the source material, fungal tissue, extraction method and concentrations of key bioactive compounds. Such measures are necessary to improve reproducibility, interpret clinical outcomes and develop more reliable dosage recommendations [57].

### 4.3. Safety, Tolerability and Product Quality

*H. erinaceus* has a long history of use as food and is generally considered well tolerated at commonly studied doses. Available clinical and toxicological evidence indicates that adverse effects are uncommon and usually mild, although long-term human safety data are still limited [1,58]. Product quality is important because commercial preparations may vary in mushroom material, cultivation conditions, extraction method and level of standardisation. These differences can affect the concentrations of bioactive compounds and may partly explain inconsistent findings between studies. Therefore, clear product characterisation and quality control are important when evaluating the safety and potential effects of supplementation [13,33]. Although supplementation is generally considered safe and well tolerated, its natural origin does not completely eliminate the possibility of adverse reactions. In the small human studies available, reported symptoms have usually been mild and have mainly included abdominal discomfort, nausea and diarrhoea. Rare hypersensitivity reactions have also been reported, so individuals with known mushroom allergies should use these products with caution [59]. Current toxicological findings are reassuring, although they are largely based on preclinical research.

Mahadevan et al. [58] evaluated an organic powder in acute, subchronic and genotoxicity studies in animal models and found no treatment-related adverse effects under the tested conditions. However, animal findings cannot be directly translated into long-term safety recommendations for humans because product formulations, exposure conditions and biological responses may differ. Human safety data are also short-term and based on small samples. In a pilot study involving 13 healthy adults, consumption of 3 g per day of powder for seven days did not significantly affect routine haematological parameters or the liver enzymes AST and ALT [27]. Similarly, according to LiverTox, no cases of clinically apparent liver injury attributed to this mushroom have been identified in the small clinical studies currently available. However, it also noted that large prospective safety trials are lacking [60]. Potential drug interactions should also be considered, although the available evidence is largely derived from preclinical studies. Experimental data indicate antiplatelet activity of *H. erinaceus* constituents, particularly hericenone B, which inhibits collagen-induced platelet aggregation [60]. In addition, hypoglycaemic effects of *H. erinaceus* polysaccharides have been reported in experimental models of impaired glucose metabolism [61]. These findings suggest that caution may be appropriate for individuals receiving antiplatelet or anticoagulant medications because of a potential additive effect on bleeding risk, as well as for those receiving antidiabetic medications because of the potential for altered blood glucose levels. Clinical evidence regarding such interactions remains limited, and further human studies are needed to clarify their relevance to *H. erinaceus* supplementation. Overall, the available studies do not allow firm conclusions about the safety of long-term supplementation or the risk of uncommon adverse effects. Larger and longer human studies should use standardised formulations and include systematic monitoring and reporting of adverse events to establish safe dosage ranges in different populations [58].

## 5. Future Directions for Research

Future research should include larger randomised, double-blind, placebo-controlled clinical trials. Studies should involve clearly defined groups, such as healthy younger adults, older adults, people with mild cognitive impairment and patients with neurodegenerative disorders. This would help determine whether the effects of supplementation differ by age, cognitive status or stage of disease [1,36]. Studies should also last long enough to assess both short-term and long-term effects. Different doses should be compared, and participants should be followed after supplementation ends. In addition to cognitive tests, future studies should consider measuring biomarkers such as BDNF, inflammatory markers, indicators of oxidative stress and gut microbiota composition. This could help explain how the supplement may affect brain function [1,37]. It is also important to use clearly described and standardised products. Researchers should state whether a product contains fruiting body, mycelium or an extract and report the extraction method and concentrations of key bioactive compounds. Analytical methods can confirm product identity and ensure that different batches have similar composition [53]. Future research should also examine the bioavailability and metabolism of compounds such as erinacine A, because the amount present in a supplement may not be the same as the amount available in the body after consumption [55]. Finally, long-term safety needs more careful study. Clinical trials should report adverse effects consistently, include laboratory monitoring when needed and use longer follow-up periods. This would help determine whether *H. erinaceus* can provide clinically meaningful health benefits and support the development of evidence-based recommendations for supplementation. The main potential benefits and current limitations of *H. erinaceus* supplementation are summarised in Table 2.

## 6. Conclusions

*H. erinaceus* is an edible and medicinal mushroom that has attracted growing interest because of its potential effects on cognitive function, neuroprotection, mental well-being and healthy ageing. Its bioactive compounds, particularly hericenones and erinacines, may influence neurotrophic signalling, oxidative stress, inflammation and neuronal survival. Experimental studies and some clinical trials have reported beneficial effects on selected outcomes, particularly processing speed, stress and other aspects of cognitive function. Current evidence remains limited and inconsistent. Many reported effects are based on cell and animal studies, while human clinical trials are relatively few, small and often short in duration. Differences in the type of supplement, its composition, dosage and study design also make it difficult to compare results and draw firm conclusions. Although supplementation appears to be generally well tolerated, long-term safety and optimal dosage have not yet been fully established.

At present, the available evidence suggests that *H. erinaceus* may have potential as a complementary approach for supporting brain health, but its clinical effectiveness has not yet been established. Larger and longer clinical studies using well-characterised and standardised products are needed to clarify the clinical relevance of the observed effects, determine the contribution of proposed mechanisms of action and support reliable recommendations for safe supplementation.

## Figures and Tables

**Table 1:** Summary of human clinical studies evaluating *Hericium erinaceus* supplementation published between 2021 and 2026.

Study	Population/Sample Size	Product/Preparation	Dose	Duration	Main Outcomes	Key Results
Xie et al. (2021) [30]	Healthy adults; *n* = 13	*H. erinaceus* powder from submerged culture	3 g/day	7 days	Gut microbiota; serum biochemical and haematological parameters	Increased gut microbiota diversity and altered serum parameters.
Grozier et al. (2022) [31]	College-age adults; *n* = 24	*H. erinaceus* extract	10 g/day	4 weeks	Metabolic flexibility; cognition	No significant effects on metabolic flexibility or cognition.
Chan et al. (2022) [32]	Older adults with hearing impairment; *n* = 80 enrolled	Erinacine A-enriched *H. erinaceus* mycelia	2 g/day	8 months	Hearing function; NGF; BDNF	Improvements in hearing outcomes; increased NGF in older participants.
Docherty et al. (2023) [33]	Healthy young adults; *n* = 41	*H. erinaceus* mushroom complex	1.8 g/day	28 days	Cognition; stress; mood	Improved acute processing speed; mixed chronic effects.
Černelič Bizjak et al. (2024) [34]	Healthy older adults; *n* = 33	Erinacine A-enriched *H. erinaceus* preparation	3 g/day	8 weeks	Cognitive performance; BDNF; NPY; gut microbiota	Improved cognitive processing speed; increased BDNF.
Surendran et al. (2025) [35]	Healthy young adults; *n* = 18	Standardised *H. erinaceus* fruiting-body extract	3 g/day	Acute; assessment at 90 min	Executive function; working memory; attention; psychomotor skills; mood	No overall cognitive or mood effect; improvement in one task.
Daoust et al. (2026) [36]	Adults with subjective cognitive difficulties; *n* = 109	*H. erinaceus* fruiting body and mycelial biomass	2 g/day	8 weeks	Cognition; sleep; subjective well-being	Improvements in selected cognitive, sleep and well-being measures.

**Table 2:** Summary of the potential benefits and main limitations of *Hericium erinaceus* supplementation.

Aspect	Potential Benefits	Main Limitations	Key References
Cognitive function	May improve memory, attention and processing speed	Clinical evidence remains limited and inconsistent	[1,30,31]
Neuroprotection	Supports nerve growth factor (NGF) and brain-derived neurotrophic factor (BDNF) signalling, neuroplasticity and neuronal survival	Most evidence is derived from preclinical studies	[9,12,36,38]
Oxidative stress	Antioxidant activity may protect neurons from oxidative damage	Human evidence is still limited	[3,36,57]
Inflammation	Anti-inflammatory properties may contribute to neuroprotection	Mechanisms require further clinical confirmation	[3,13,18]
Gut health	May modulate gut microbiota and support gastrointestinal function	Clinical significance remains unclear	[5,27]
Safety	Generally well tolerated in short-term supplementation	Long-term safety and optimal dosage are unknown	[58,59]
Product quality	Multiple bioactive compounds with potential biological effects	Lack of standardisation and variability among commercial products	[53,54,55,56,57,58]
Clinical application	Potential complementary approach for healthy ageing	Insufficient evidence for routine clinical use	[1,36,43]

## Data Availability

No new data were created or analysed in this study. Data sharing is not applicable to this article.
